# Polypills in the Management of Cardiovascular Risk—A Perspective

**DOI:** 10.3390/jcm13185487

**Published:** 2024-09-16

**Authors:** Erlon Oliveira de Abreu-Silva, Martin Siepmann, Timo Siepmann

**Affiliations:** 1 Hcor Research Institute, Hcor (IP-Hcor), Abílio Soares Street 250, São Paulo 04004-050, Brazil; 2 Division of Health Care Sciences, Dresden International University, Freiberger Str. 37, 01067 Dresden, Germany; timo.siepmann@ukdd.de; 3 Department of Psychotherapy and Psychosomatic Medicine, Faculty of Medicine, TUD Dresden University of Technology, Fetscherstrasse 74, 01307 Dresden, Germany; martin.siepmann@ukdd.de; 4 Department of Neurology, Medical Faculty and University Hospital Carl Gustav Carus, TUD Dresden University of Technology, Fetscherstrasse 74, 01307 Dresden, Germany

**Keywords:** cardiovascular disease, polypill, mortality, blood pressure, LDL-cholesterol, adherence

## Abstract

Cardiovascular disease and cardiovascular risk factors are global healthcare problems, given their high prevalence and the recognized low rates of adequate control despite the abundant body of evidence on different therapeutic options. The World Heart Federation has scrutinized the reasons for poor control of cardiovascular risk factors. Among these reasons, patients’ poor adherence to treatment regimens as well as limited rates of evidence-based therapy prescription from healthcare providers play a substantial role in the challenge of cardiovascular risk management. Polypills are fixed-dose combinations including two or more active drugs, from different pharmacological classes, combined in a single dosage form. Polypills were designed to simplify the clinical management of pharmacotherapy and increase adherence to treatment. From this perspective, we discuss the current literature on the use of polypills in the primary and secondary prevention of cardiovascular disease as well as future challenges and the potentials of this treatment strategy.

## 1. Introduction

Cardiovascular disease (CVD) is the leading cause of mortality worldwide [1]. Hyperlipidemia [2], hypertension [2,3] and diabetes mellitus [3] are all examples of conditions associated with the development of CVD, the so-called cardiovascular risk factors. These risk factors are characterized by distinct pathophysiology, natural history and treatment. They have in common that they worsen the atherosclerotic process. It can be asserted that cardiovascular risk factors constitute individual diseases that lead to cumulative damage of the heart and vasculature, eventually increasing the risk of devastating events like myocardial infarction (MI), stroke or cardiovascular death. On the other hand, modifiable cardiovascular risk factors can lead to relevant organ damage, like brain damage, even in young healthy adults before the time any cardiovascular disease is diagnosed [4]. Viewed in conjunction with the substantial variance in rates of successful management of cardiovascular risk factors, it becomes apparent that adequate control of cardiovascular risk factors remains one of the pivotal challenges of global healthcare. 

Indeed, despite the documented benefits of available treatments, the rates of adequate control are far from optimal. An analysis of the National Health and Nutrition Examination Survey study showed that, between 2017 and 2018, in the United States, 43.7% of those with hypertension had controlled blood pressure levels [5]. According to an analysis of the World Health Organization from 2023, this number drops to 21% at a global scale [6]. In a pooled analysis of population-level studies, 41.9% of participants had target levels of HbA1c [7]. Control of low-density lipoprotein-cholesterol (LDL-c) was seen in 49.3% of subjects overall, but only in 26.4% among those with established CVD [8]. 

Aligned with the World Health Organization Global Action Plan for the Prevention and Control of Non-Communicable Diseases, the World Heart Federation (WHF) suggested a roadmap for the optimization of healthcare in CVD [9]. This roadmap pays special attention to the prescription and use of medications with known efficacy in reducing cardiovascular events (namely, acetylsalicylic acid (ASA), statins and angiotensin-converting enzyme inhibitors (ACEI)/angiotensin receptor blockers (ARBs)). It also includes recommendations of strategies to facilitate lifestyle modification (smoking cessation, for example), alongside patient adherence. Poor adherence has been suggested to reduce the effects of preventive measures in various populations [9]. 

That statement indicated potential roadblocks and proposed potential solutions. For situations in which recommendations on drug treatment are too complex or there is evidence of poor adherence by patients, it is recommended to use fixed-dose combinations of medications with known cardiovascular benefit [9].

The concept of a fixed-dose combination of different pharmacological classes to be taken in a single daily dose, a polypill, capable of controlling risk factors and reducing cardiovascular risk, has been tested in both primary and secondary prevention. It has shown significant reduction in blood pressure (BP), LDL-c levels, and cardiovascular events, including death [10,11,12,13,14,15,16,17,18,19,20,21,22,23,24,25,26,27,28,29]. These results have been translated to formal recommendations of a polypill in recently published guidelines [30,31]. 

From this perspective, we revisit the main studies supporting the evidence for the use of a polypill-based strategy to control of cardiovascular risk factors and reduce cardiovascular risk. Moreover, we discuss possible challenges ahead.

## 2. Methods

This is a perspective following the methods of a narrative review to present the state-of-the-art and current body of knowledge regarding the use of polypills in the context of reducing events and cardiovascular safety. We conducted a literature search, between 10 January and 10 May, 2024, using the PubMed and Scopus databases, irrespective of language or year of publication, employing the following keywords to be searched in the title: “polypill” AND “cardiovascular”; “polypill” AND “safety”. Randomized clinical trials and meta-analyses were the prioritized study designs included, although clinical guidelines were also within the scope for citation. Studies with a higher number of citations, particularly those cited by society guidelines, and those included in systematic reviews or meta-analyses were selected.

## 3. Aspirin, Antihypertensive Drugs and Statins for the Prevention of Cardiovascular Events—Evidence from Individual Trials

The analysis of the impact of CVD and its risk factors makes the importance of risk stratification obvious, since previous studies have already demonstrated that the level of non-controlling of these risk factors and/or the baseline risk is directly related to potential benefit of adequate management [32,33].

Guidelines from several entities around the world recommend the use of medications with documented benefit in reducing cardiovascular events, with emphasis on ASA, ACEI/ARBs and statins [30,34,35,36,37]. 

In subjects with established atherosclerotic disease, the use of ASA is associated with a significant reduction in severe vascular events, including stroke and coronary events, and with a 10% lower total mortality rate [38]. These benefits outweigh the risk of bleeding [38]. 

Among those with coronary artery disease (CAD), ASA is indicated for all cases after an acute coronary syndrome (ACS), revascularization procedures or with chronic coronary syndrome (CCS) and no indication for oral anticoagulation [30,34,37,38]. The use of ASA may also be considered in individuals with evidence of CAD in image exams or in persons with diabetes and high/very-high cardiovascular risk [34]. 

Despite the marked benefits in those in higher risk strata, ASA is not indicated for those at low and moderate risk [34]. 

In patients with ischemic stroke or transient ischemic attack (TIA), using antithrombotic drugs prevents new vascular events [38]. When the mechanism of the cerebrovascular event is embolic, as in people with atrial fibrillation, anticoagulation is indicated. In cases of non-embolic ischemic strokes, ASA is the most studied antithrombotic and is indicated to reduce the risk of a recurrent ischemic stroke and severe vascular events [34,38].

Antiplatelet therapy is used to prevent limb-related and cardiovascular events (death, MI, stroke) in those with peripheral artery disease (PAD) [39] and is indicated for symptomatic cases [34,35]. 

The benefits of ACEI and ARBs in the treatment of hypertension and cardiovascular risk reduction in persons with high risk, such as those with diabetes, CCS, heart failure (HF), PAD and previous ACS or stroke, have already been demonstrated [40,41,42,43,44,45]. The blockade of the renin-angiotensin-aldosterone system (RAAS) improved symptoms and reduced hospitalizations and mortality in the different manifestations of CAD, independently of the left ventricle ejection fraction (LVEF) [40,41,42,43,44,45]. Additionally, it reduced micro- and macrovascular outcomes in persons with diabetes and recurrency of stroke [40,41,42,43,44,45]. 

In individuals with CCS and who also present with hypertension, diabetes, LVEF ≤ 40% or chronic kidney disease (CKD), their use is formally indicated [34,37], such as for those recovering from an ACS [30].

The use of ACEI/ARBs has been shown to reduce the recurrence of ischemic stroke and TIA [44,45] and have the preferential indication in the guidelines [34] with special attention on BP control. They are also the preferred BP-lowering drugs for those with PAD [35].

Lastly, both classes are in the center of the HF treatment algorithm, alongside beta-blockers, mineralocorticoid antagonists and SGLT2 inhibitors [36]. 

The use of statins, on the other hand, reduced cardiovascular events in persons with diabetes [46], even among those out of the higher-risk strata according to the Global Risk Score (GRS) [47]. In patients with high and very-high cardiovascular risk, such as those with established CVD, in secondary prevention, with CCS or PAD, the use of a high-potency regimen of statin was capable of a more aggressive LDL-c reduction and lower rates of cardiovascular events [33,48], and its use is formally indicated in these clinical scenarios [30,34,35,37].

## 4. Polypill-Based Strategies in Cardiovascular Risk Management

Wald and Law claim to have introduced the concept of a formulation for the prevention of cardiovascular disease [49] based on the following principles: a large preventive effect would require intervention in everyone at increased risk, irrespective of the risk factor levels; intervention on several reversible causal risk factors together; and reducing these risk factors by as much as possible [50].

This polypill was composed of a statin, three antihypertensive drugs, ASA and folic acid and was aimed at lowering LDL-c, BP, platelet function and homocysteine levels [49,51].

In their assay, they used an intricate mathematical model to multiply the relative risks associated with each risk factor to calculate the combined effect of changing the four risk factors (i.e., the effect of the polypill) based on efficacy data from randomized trials and meta-analyses of its individual components [51]. In that assumption, they estimated a reduction of 88% in CAD events (95% confidence interval—CI—84% to 91%) and of 80% in stroke (95%CI 71% to 87%) [51]. Interestingly, the estimated rate of symptoms attributable to the polypill varied from 8 to 15%, depending on the specific formulation [51].

Ever since, the effect of a combination of different guideline-recommended pharmacological classes to reduce cardiovascular risk, in a single and fixed-dose, has been tested in different parts of the globe, with various combinations, in people with and without established CVD [10,11,12,13,14,15,16,17,18,19,20,21,22,23,24,25,26,27,28,29].

### 4.1. Polypill-Based Strategies in Primary Cardiovascular Prevention 

The TIPS study evaluated the effect of a polypill made of hydrochlorothiazide 12.5 mg + ramipril 5 mg + simvastatin 20 mg + ASA 100 mg with some of these components, isolated or combined in different formulations, in 2053 adults aged 45 to 80 years with one risk factor, but without CVD, enrolled from 50 centers in India [10]. After 12 weeks, the group receiving the polypill had significant reductions in BP and LDL-c compared to those who were not on anti-hypertensive medications or statins, respectively [10]. The authors theorized, based on their results and using the same mathematical model as Wald and Law [51], that the reductions seen on TIPS could translate into a reduction in the risk of CAD and stroke of 62% and 48%, respectively [10]. 

A combination of hydrochlorothiazide 12.5 mg + enalapril 2.5 mg + atorvastatin 20 mg + ASA 81 mg was compared to placebo in 475 adults in Iran aged between 50 and 79 years with no previous diagnosis of hypertension, hyperlipidemia or CVD [11]. During the 1-year follow-up, there was a significant reduction in BP and LDL-c levels (primary outcome) among those randomly allocated to the polypill group [11].

Another formulation with ASA, statin, an ACEI and a thiazide-type diuretic was tested by the PILL Collaborative Group [12]. In this study, 378 adults (minimum 18 years old) with a 7.5% or higher risk of events in 5 years, according to the Global Risk Score (GRS), were enrolled in centers from Australia, England, Brazil and The Netherlands, and were followed for 12 weeks. Primary outcomes were, once more, BP and LDL-c levels that were significantly reduced in the group who used the polypill (hydrochlorothiazide 12.5 mg + lisinopril 10 mg + simvastatin 20 mg + ASA 75 mg) in comparison to the placebo [12]. 

Van Gils and colleagues evaluated the cost-effectiveness of a polypill-based healthcare strategy, compared to usual care (the use of different drugs—not necessarily the same as in the polypill—in their own usual formulations, presentations and posology, at the assistant physicians’ discretion), in over 1 million high-cardiovascular-risk adults (5–10% risk of cardiovascular death in 10 years, according to SCORE) aged 45 to 75 years old in the Netherlands, in a simulation study using a computer model in which eligible patients would receive a polypill once identified during routine visits to the general practitioner [13]. The outcomes were cases of prevented myocardial infarction (MI) and stroke, gained quality-adjusted life years (QALY) and costs per gained QALY. In all scenarios, the polypill-based strategy had better cost-effectiveness [13].

Wald and colleagues evaluated the effect of a formulation containing hydrochlorothiazide 25 mg + losartan 25 mg + amlodipine 2.5 mg + simvastatin 40 mg, versus placebo, on BP and LDL-c levels in adults, at least 50 years old and without known CVD, in London [15]. A total of 86 subjects were followed for 12 weeks taking the polypill and 12 weeks taking placebo in a crossover design. Using the polypill was associated with significantly lower BP levels (systolic BP/diastolic BP −12%/−11%) and lipid profile levels (LDL-c: −39% and triglycerides—TG—−23%) [15]. 

The effect of a fixed-combination of hydrochlorothiazide 12.5 mg + losartan 25 mg + amlodipine 2.5 mg+ Atorvastatin 10 mg was tested against usual care in 303 adults in situations of vulnerability, without known CVD but with a mean risk of 12.7% of cardiovascular events in 10 years, according to the American Heart Association/American College of Cardiology (AHA/ACC), meaning intermediate cardiovascular risk, in the state of Alabama, USA [24]. The annual income of ¾ of the sample was under USD 15,000; 96% were black and, at the end of a 12-month follow-up, there was a significant reduction in systolic BP and LDL-c levels among those allocated to the polypill group, at a monthly cost of USD 26 [24]. 

In the randomized, factorial 2 × 2, clinical trial TIPS 3, the authors evaluated the combination of hydrochlorothiazide 25 mg + ramipril 10 mg + simvastatin 40 mg + atenolol 100 mg versus placebo, and ASA 75 mg versus placebo, in primary prevention (subjects without known CVD, but with an at least moderate cardiovascular risk according to the Interheart score) [25]. A total of 5713 subjects were enrolled, with a mean follow-up of 4.6 years, and the results showed that the primary outcome, a composite of cardiovascular death, MI, stroke, cardiac arrest, HF or arterial revascularization occurred in 4.4% of those allocated to the polypill versus 5.5% of those receiving placebo (hazard ratio—HR—0.79; 95%CI 0.63–1.0), and in 4.1% among those receiving the polypill + ASA vs. 5.8% among those receiving double placebo (HR 0.69; 95%CI 0.50–0.97) [25]. 

A recent meta-analysis, comprising 18,162 participants from the HOPE-3 [20], PolyIran [21] and TIPS 3 trials [25], has compared the time until the composite outcome of cardiovascular death, MI, stroke or arterial revascularization, stratifying data by the presence, or not, of ASA in the different polypill preparations tested in primary prevention [26]. The results showed that, after a mean follow-up of 5 years, a polypill-based strategy had less events (3.0% versus 4.9%; HR 0,62, 95%CI 0.53–0.73; *p*< 0.001), and this reduction was significant independently of the presence of ASA [26]. Nevertheless, among those who used ASA, this reduction was more pronounced, even though there was an increased rate of gastrointestinal (GI) bleeding (*p* = 0.15) [26]. 

More recently, the VULCANO study randomized adults with high and very-high cardiovascular risk, but with no previous cardiovascular event, to a formulation of ramipril 2.5, 5 or 10 mg + atorvastatin 20 or 40 mg + ASA 100 mg versus usual care for 16 weeks and demonstrated non-inferiority and superiority of the polypill in reducing LDL-c levels, but there was no difference in BP control [28].

Lastly, a meta-analysis of 11 trials comprising 17,042 subjects evaluated the effect of different polypill formulations on BP levels [29]. The results have shown a better BP control compared to usual care [29]. 

### 4.2. Polypill-Based Strategies in Secondary Cardiovascular Prevention

The TIPS-2 study compared two different dosages (1 versus 2 capsules) of a fixed-combination of hydrochlorothiazide 12.5 mg + atenolol 50 mg+ ramipril 5 mg + simvastatin 20 mg + ASA 75 mg in 518 people, 40 years old or more, with multiple cardiovascular risk factors or CVD, recruited from 27 Indian centers [14]. More than half the sample had CAD, 12.5% had a history of cerebrovascular disease, 1.7% had PAD and 40.9% had diabetes. After 8 weeks of follow-up, the higher dose achieved significantly lower levels of BP and LDL-c, with similar tolerability compared to the lower dosage [14]. 

The UMPIRE trial compared the use of a polypill to standard care in patients at high cardiovascular risk [16]. This randomized clinical trial enrolled 2004 adults (minimum age: 18 years old) categorized as high cardiovascular risk (established CVD or estimated risk in 5 years ≥15% according to Guidelines from New Zealand). Patients were randomized to either receive a polypill (lisinopril 10 mg + simvastatin 40 mg + ASA 75 mg + hydrochlorothiazide 12.5 mg or atenolol 50 mg) or usual care [16]. At the end of a mean follow-up of 15 months, the group receiving the polypill displayed higher adherence (86% vs. 65%; relative risk—RR—1.33 95%CI 1.26–1.41; *p* < 0.001; in absolute terms, a 21.6% difference, with a number needed to treat (NNT) of 4.6); lower BP (−3.3 mmHg, 95%CI −4.6 a −1.9; *p* < 0.001) and LDL-c (−5.3; 95%CI −7.5 a −3.2; *p* < 0.001). This effect was more pronounced among those with poor adherence at baseline [16].

Castellano and colleagues compared adherence and efficacy of a ramipril 2.5 or 10 mg + simvastatin 40 mg + ASA 100 mg polypill, using the three medications separately in post-MI subjects in centers from Italy, Brazil, Paraguay and Spain [17]. A total of 695 individuals, with ages of 40 years or more, was followed for 9 months, and those using the polypill had better adherence (Morisky score = 20; 50.8% vs. 41%; *p* = 0.019—ITT; 65.7% vs. 55.7%; *p* = 0.012), with no difference in BP, LDL-c, adverse events or death [17]. Selak and colleagues, on the other hand, conducted a randomized trial with 513 high-cardiovascular-risk (established CVD or estimated risk in 5 years ≥15% according to Guidelines from New Zealand) adults (257 Maori), in Oceania, and compared a polypill (lisinopril 10 mg + a 40 mg + ASA 75 mg + hydrochlorothiazide 12.5 mg or atenolol 50 mg) with the use of these 4 drugs separately [18]. By the end of the minimum follow-up of 12 months, the polypill group had better adherence (81% vs. 46%; RR 1.75 95%CI 1.52–2.03; *p* < 0.001; number needed to treat (NNT), 2.9), without difference in BP or LDL-c [18]. This same polypill formulation was tested against usual care in 623 adults, in the same cardiovascular risk stratum, in Oceania, and was followed for 36 months by Patel and colleagues [19]. The results showed that, compared to the group who used the medications separately, those in the polypill group had better adherence (70% vs. 47%, RR 1.49; IC95% 1.30–1.72; *p* < 0.001), but there was no difference in BP or total cholesterol (TC) [19]. 

Roshandel and colleagues conducted a cluster randomized study in Iran with approximately 7000 adults aged between 40 and 75 years old and evaluated the effect of a polypill made of hydrochlorothiazide 12.5 mg + enalapril 5 mg + atorvastatin 20 mg + ASA 81 mg versus orientations regarding lifestyle modifications and usual care in primary and secondary cardiovascular prevention [21]. Those who received the polypill had a significant reduction in the primary composite outcome of hospitalization for acute coronary syndrome (ACS), sudden death, non-fatal MI, HF, coronary revascularization and fatal and non-fatal stroke (HR 0.66; 95%CI 0.55–0.8), without significant interaction with the presence or absence of known CVD (with CVD: HR 0.61; 95%CI 0.49–0.75; without CVD: HR 0.8; 95%CI 0.51–1.12; *p* = 0.19) [21]. 

A meta-analysis of the UMPIRE [16], IMPACT [18] and Kanyini-GAP [19] studies synthesized data from 3410 subjects, 76% of whom had a known previous cardiovascular event, and evaluated the percentage of persons with BP and LDL-c levels within the recommended targets, according to the European Society of Cardiology (ESC) guidelines, and in the use of antiplatelet aggregation between the polypill-based strategy and usual care [22]. The analysis showed that, after 12 months using a polypill-based treatment, compared to usual care, the relative risk (RR) of controlling BP, LDL-c and all three parameters were 1.08 (1.02–1.15), 1.13 (1.02–1.25) and 1.27 (1.1–1.47), respectively [22].

In Central America, Gómez-Álvarez and colleagues evaluated 572 people with established CVD before and after using a polypill of ramipril 5 or 10 mg + simvastatin 40 mg + ASA 100 mg, in a unicentric study in Mexico [23]. After 12 months using the polypill, the rate of BP control rose from 20.1% to 55.4%, compared to previous standard care (*p* < 0.001) [23].

The most relevant evidence, so far, regarding the use of a polypill-based strategy in secondary cardiovascular prevention comes from the SECURE trial [27]. 

This study evaluated the effect of a combination of ramipril 2.5, 5 or 10 mg + atorvastatin 20 or 40 mg + ASA 100 mg compared to usual care (according to ESC guidelines), in adults with a recent MI (in the previous 6 months) and aged, at least, 75 years old, or adults 65 years old or more with one of the following additional diagnoses: diabetes, chronic kidney disease (estimated glomerular filtration rate (eGFR) between 30 and 60 mL/min), previous MI (besides the index event), previous coronary revascularization or stroke [27]. The trial enrolled a total of 2499 subjects in centers from the Czech Republic, Spain, Italy, France, Germany, Poland and Hungary, and had a composite of cardiovascular death, non-fatal MI (type 1), non-fatal stroke and urgent arterial revascularization as the primary outcome [27]. Median follow-up was 3 years; almost 99% of the participants were white. 

There was a significant reduction in the rates of the primary outcome in the group randomized to the polypill, compared to usual care (9.5% versus 12.7%; HR 0.76 95%CI 0.6–0.96; *p* = 0.02) [27]. It is noteworthy that the individual components of the composite primary outcome, including cardiovascular death, were also significantly less frequent among those receiving the polypill. Adherence was higher in those allocated to the polypill group (RR 1.13; 95%CI 1.06–1.20), with no excess of adverse events [27]. 

## 5. Safety of Polypill-Based Strategies for Cardiovascular Risk Reduction 

One of the potential disadvantages of using a single pill with various medications is the occurrence of adverse effects. Therefore, safety has been a crucial part of the research on polypills. 

In the TIPS study, the polypill (hydrochlorothiazide 12.5 mg + ramipril 5 mg + simvastatin 20 mg + ASA 100 mg) was as well tolerated as the controls (some of these components, isolated or combined in different formulations) [10]. The same was seen in the comparison between the combination of hydrochlorothiazide 12.5 mg + enalapril 2.5 mg + atorvastatin 20 mg + ASA 81 mg and placebo in the study by Malekzadeh and colleagues [11]. 

The PILL Collaborative Group compared a combination of hydrochlorothiazide 12.5 mg + lisinopril 10 mg + simvastatin 20 mg + ASA 75 mg to placebo in primary prevention in subjects at increased cardiovascular risk and reported a significantly higher rate of side effects among those allocated to the active treatment (58% vs. 42%, *p* = 0.001) [12]. The excesses were mostly due to the well described effects of ASA (gastric irritation and/or bleeding tendency in 17% of the active group vs. 6% of placebo) and of ACEI-based antihypertensive therapy (cough and/or lightheadedness, dizziness or hypotension in 30% vs. 11% of the active and placebo groups, respectively). Interestingly, the rates of treatment discontinuation were not different through the 12-week follow-up (23% in the polypill vs. 18% in placebo, *p* = 0.2) [12]. 

The TIPS-2 study compared two different dosages (1 versus 2 capsules) of a fixed-combination of hydrochlorothiazide 12.5 mg + atenolol 50 mg+ ramipril 5 mg + simvastatin 20 mg + ASA 75 mg in secondary prevention [14]. Overall rates of any discontinuation during the follow-up were 11.9% in the low dose group and 14% in the full dose group. The only specific reason for discontinuation in which there was a significant difference was dyspepsia, with overall reported rates of 0.4% in the low dose group and 3.5% in the full dose group (*p* = 0.01). Considering only those who permanently discontinued the study medications, dyspepsia was the reason for it in 0.4% in the low dose group and 2.7% in the full dose group [14]. Noteworthy is the fact that, as it could be expected in a trial where BP should be ≥130/80 mmHg (or ≥120/80 mmHg on antihypertensive drugs), there was no difference in rate of dizziness between groups [14]. 

In a randomized crossover trial with 86 people taking a polypill containing hydrochlorothiazide 25 mg + losartan 25 mg + amlodipine 2,5 mg + simvastatin 40 mg for 12 weeks, and placebo for an additional 12 weeks [15], even though the trial was not designed to assess side-effects of the polypill, a questionnaire on the recognized side-effects of the component medications (cough, muscle ache or pain, ankle swelling, flushing, rash, tongue and lip swelling) was completed at the end of each 12-week treatment period [15]. Symptoms were reported by 24 subjects during the polypill period and 11 subjects during the placebo period (*p* = 0.01), but with no reported need for discontinuing treatment [15].

In the UMPIRE trial, a combination of lisinopril 10 mg + simvastatin 40 mg + ASA 75 mg + hydrochlorothiazide 12.5 mg or atenolol 50 mg was compared to usual care in secondary prevention [16]. From the 1002 participants in the polypill group, 219 discontinued the treatment. The main reasons were patient choice (28%), medical advice with no specified reason or adverse event (26%), cough (21%), dizziness (9%), serious adverse events (8%), other adverse events (16%) or other reasons (8%) [16]. As can be seen, more than half of these cases were not specifically related to any adverse event and 21% were expected among ACEI users. The rates of serious adverse events were not different between the study arms [16].

A ramipril 2.5 or 10 mg + simvastatin 40 mg + ASA 100 mg polypill was tested against using the three medications separately in post-MI subjects, and there was no significant difference in adverse events [17]. A total of 35% participants taking the polypill and 32% in the control group reported an adverse event (6% vs. 6.6%, respectively, for serious adverse events) [17]. Treatment was discontinued in 4% of participants in each group [17].

In the IMPACT trial, high-risk individuals for whom the assistant physician considered that all the drugs of at least one of the formulations were recommended were randomized to receive a lisinopril 10 mg + a 40 mg + ASA 75 mg + hydrochlorothiazide 12.5 mg or atenolol 50 mg polypill or the use of the respective four drugs separately [18]. There was no significant difference in the number of participants with serious adverse events (*p* = 0.56). An excess of reported serious adverse events occurred in some categories: hypotension (polypill 6 vs. usual care 0, *p* = 0.01) and bleeding (4 vs. 0, *p* = 0.06). Presumably, due to the results of better adherence, these findings were at least in part due to the higher use of BP-lowering drugs and ASA in the polypill group [18]. This same polypill formulation was compared to usual care in high-risk adults followed for 36 months [19]. The results showed similar rates of serious adverse events (46.3% in the polypill group vs. 40.7% in usual care, *p* = 0.16). The polypill was discontinued in 29.9% of participants, and the main reasons for this were assistant physician`s decision (41.8%), patient choice (17.4%), cessation by a specialist or during hospitalization (15.1%), cough (15.1%) or dizziness/hypotension (5.8%).

In the PolyIran cluster trial, a combination of hydrochlorothiazide 12.5 mg + enalapril 5 mg + atorvastatin 20 mg + ASA 81 mg was compared to usual care, and the patterns and frequency of adverse events were similar in both groups [21]. 

In the evaluation of a fixed combination of hydrochlorothiazide 12.5 mg + losartan 25 mg + amlodipine 2.5 mg + Atorvastatin 10 mg against usual care in Alabama, USA, there were five serious adverse event that were considered not to be related to the trial procedures [24]. The reported incidences of myalgia and hypotension/lightheadedness were both 1% in the polypill group [24].

In a meta-analysis with 18,162 participants from trials on the use of different polypill preparations, with and without ASA, safety analysis considered those potentially related to statins or antihypertensive drugs and those potentially related to ASA [26]. Muscle pain was significantly more frequent among controls than among those who used a polypill (8.7% vs. 7.0%, *p* < 0.0001), while the occurrence of dizziness had an opposite behavior (9.2% vs. 11.7%; *p* < 0.0001) [26]. Considering the events potentially related to ASA, there was no significant difference between groups in either comparison [26], although the occurrence of GI bleeding was higher in the polypill group and those of hemorrhagic stroke, death due to bleeding, peptic ulcer disease or dyspepsia were higher among controls [26]. 

In a very-high-cardiovascular-risk population, with increased age as an entry criterion, adverse events were reported for 32.7% of those allocated to a polypill of ramipril 2.5, 5 or 10 mg + atorvastatin 20 or 40 mg + ASA 100 mg, and for 31.6% receiving usual care [27]. There was, also, no significant difference in the rates of non-fatal serious adverse events (19.2% in the polypill vs. 18.2% in control) [27]. 

## 6. Advantages and Concerns of Polypill-Based Strategies for Cardiovascular Risk Reduction 

The potential advantages of such polypill-based strategies were already brought to light in 2010, comprising improved delivery of care, improved adherence, reduced cost and use of the polypill platform for novel approaches to widespread CVD prevention [52]. 

A better delivery of care—through the avoidance of complex algorithms to identify eligible subjects to receive the polypill—alongside an easier prescription [52] and, ultimately, improved adherence, were seen in the studies conducted during the following years [10,11,12,13,14,15,16,17,18,19,20,21,22,23,24,25,26,27,28,29].

When it comes to safety concerns, it is important to highlight that, in some studies, hypertension was not a specific inclusion criterium [12,15,18,19]. Instead, the estimate of cardiovascular risk was. This may explain the excess of lower BP-related symptoms in some studies, as well as increased adherence [12,18,19]. Another relevant point is that, as expected, cough would be more frequent among those taking an ACEI than for those taking other antihypertensive medications as part of usual care or those who took placebo as the control comparator [12,15,16,19]. In both cases, the concept of the polypill allows the choice of the best components for each patient, as well as dose titration, to optimize the benefits and minimize the risks. 

Although evaluations of costs related to a polypill-based strategy have demonstrated promising results [13,24,53,54], some remarks are noteworthy.

A simulation study analyzed a hypothetical scenario of optimized use of a polypill (combination of three antihypertensive drugs, ASA and statin) in routine healthcare in The Netherlands, a high-income country (HIC), and the strategy was shown to be more cost-effective than usual care [13] in primary cardiovascular prevention, at an annual cost of EUR 89.75. In a study conducted in the USA, another HIC, but in a setting of primary prevention among underserved people, the use of a polypill with three BP-lowering medications plus a statin was beneficial and cost USD 26/month (USD 312/year). 

Lin and colleagues [53] developed microsimulation models to assess the cost-effectiveness of a polypill containing two antihypertensive drugs, ASA and a statin for secondary prevention compared to usual care in China, India, Mexico, Nigeria and south Africa, all low and middle-income countries (LMICs). Considering that, in these countries, medication costs can have a large variation depending on whether people obtain them through public health service or retail market, and assuming the polypill would cost the same as the sum of its individual components, the authors presented both analyses. The annual cost of the polypill, in the public sector, varied from Int$ 32.40 (Mexico) to Int$ 80 (India) [53]. In the retail market, annual costs varied from Int$ 239.20 (India) to Int$ 1188 (China) [53]. 

An analysis of the TIPS-3 trial evaluated the potential costs of a polypill with three antihypertensive drugs and a statin, with and without ASA, in LMIC (Bangladesh, India, Tunisia and the Philippines), upper-middle income countries (UMIC; Colombia, Indonesia and Malaysia) and Canada (HIC) [54]. Pricing of the polypill was based on the cheapest equivalent substitute (CES), once the specific formulation used in the trial is not marketed in any country. Considering the mean follow-up period of the trial (4.6 years), the mean cost of the formulation without ASA, for that period was USD 361 in the LMICs, USD 1195 in the UMICs and USD 327 in the HIC. On the other hand, the polypill with ASA had a mean cost of USD 395 in the LMIC, USD 1231 in the UMIC and USD 361 in the HIC, for the 4.6-year-follow-up [54]. 

Lamy and colleagues concluded that a high-potency statin (rosuvastatin) and a fixed-dose combination of two BP-lowering medications (candesartan and hydrochlorothiazide) was a cost-saving approach for primary cardiovascular prevention in developed countries, but not in developing countries because both drugs and their CES were relatively more expensive despite adjustment by gross domestic product (GDP) [55]. 

These data may raise some questions: (1) Why does this strategy seems to be cheaper in high-income areas? (2) Are the differences seen in pricing between LMIC and UMIC generalizable for all such regions across the globe or do those findings [54] portray a specific scenario for those countries? (3) Is it possible for any healthcare strategy to be cost-effective in developing countries without the logistical and financial support of the public health system? 

Other important aspects to be considered are those related to pharmaceutical formulation issues, the composition of the “ideal” polypill and registration of new formulations [52]. All these steps are necessary for the development and marketing of a new drug. This, however, comes with a high financial cost with protocols in basic, translational and clinical research that are conducted over considerable amounts of time and for which there is no guaranteed result. So far, only a few small companies have manufactured and marketed polypills in some countries. 

It is possible to assume that if larger companies invested in mass production and marketing of a polypill, this could increase the reach of this therapeutic strategy, making it more affordable, especially for underserved populations, initiating a virtuous cycle of larger supply—to match an already large and increasing demand—leading to lower cost, and so forth. 

Although the concept of a polypill may fit an approach in which healthcare delivery would be more personalized, to the best of our knowledge, there are no substantial data published on the use of a polypill for cardiovascular risk reduction in conjunction with pharmacogenetics or pharmacogenomics. This will likely pose one of the crucial challenges of polypills in the future. 

## 7. Conclusions

Considering the available evidence, the use of a polypill seems to be a useful strategy to facilitate the prescription of guideline-directed medical therapy, increase rates of adequate control of cardiovascular risk factors and decrease cardiovascular mortality, with the possibility of a lower costs, compared to usual care, in a wide range of people at increased cardiovascular risk. Future research may focus on formulations considering the inherent differences among individuals and populations, while local and global healthcare stakeholders should work on making this technology more affordable and more widely available, especially in developing countries. However, further head-to-head comparisons of both the efficacy and safety of polypill versus monopill strategies are warranted, both in a prospective interventional and a post-marketing observational setting. 
